# Ectopic Activation of Wnt/β-Catenin Signaling in Lens Fiber Cells Results in Cataract Formation and Aberrant Fiber Cell Differentiation

**DOI:** 10.1371/journal.pone.0078279

**Published:** 2013-10-30

**Authors:** Barbora Antosova, Jana Smolikova, Romana Borkovcova, Hynek Strnad, Jitka Lachova, Ondrej Machon, Zbynek Kozmik

**Affiliations:** 1 Institute of Molecular Genetics, Academy of Sciences of the Czech Republic, Prague, Czech Republic; 2 Department of Genetics and Microbiology, Faculty of Science, Charles University in Prague, Prague, Czech Republic; National Eye Institute, United States of America

## Abstract

The Wnt/β-catenin signaling pathway controls many processes during development, including cell proliferation, cell differentiation and tissue homeostasis, and its aberrant regulation has been linked to various pathologies. In this study we investigated the effect of ectopic activation of Wnt/β-catenin signaling during lens fiber cell differentiation. To activate Wnt/β-catenin signaling in lens fiber cells, the transgenic mouse referred to as αA-CLEF was generated, in which the transactivation domain of β-catenin was fused to the DNA-binding protein LEF1, and expression of the transgene was controlled by αA-crystallin promoter. Constitutive activation of Wnt/β-catenin signaling in lens fiber cells of αA-CLEF mice resulted in abnormal and delayed fiber cell differentiation. Moreover, adult αA-CLEF mice developed cataract, microphthalmia and manifested downregulated levels of γ-crystallins in lenses. We provide evidence of aberrant expression of cell cycle regulators in embryonic lenses of αA-CLEF transgenic mice resulting in the delay in cell cycle exit and in the shift of fiber cell differentiation to the central fiber cell compartment. Our results indicate that precise regulation of the Wnt/β-catenin signaling activity during later stages of lens development is essential for proper lens fiber cell differentiation and lens transparency.

## Introduction

Mouse lens morphogenesis begins with the formation of a lens placode in the head surface ectoderm in response to inductive signals from several tissues including the underlying optic vesicle. The lens placode subsequently invaginates and gives rise to the lens pit and then to the lens vesicle composed of epithelial cells, forming a single layer of cuboidal epithelium on the anterior lens pole, and fiber cells which form the remainder of the lens. Primary fiber cells differentiate from cells comprising the posterior part of the lens vesicle. Secondary fiber cells are continually generated in the equatorial region of the lens. There, the transitional zone is formed, and multiple signaling molecules converge on lens precursors, cells exit the cell cycle, and begin to elongate and differentiate into secondary fiber cells [1]. Differentiation of lens fiber cells is characterized by a change of the cell shape and by accumulation of fiber cell-specific proteins, β- and γ-crystallins, which are the major structural, water soluble lens proteins responsible for the optical properties of the lens [2]. Transcription factors Pax6, Prox1, c-Maf, and Sox1 are essential regulators of fiber cell differentiation, since they regulate expression of crystallins and their loss results in arrest of fiber cell differentiation [3]–[10]. Cell cycle inhibitors p27^Kip1^ and p57^ Kip2^ [4] are required in lens cells for the cell cycle exit at the transitional zone and for terminal differentiation and elongation of lens fiber cells, as they inhibit cyclin-dependent kinases involved in G1/S transition [11]. Fiber cell differentiation is completed by the loss of intracellular organelles and nuclei, which ensures the lens transparency [12].

Beyond transcription factors, several growth factors have been implicated as regulators of lens fiber cell differentiation. Fibroblast growth factors (FGFs) [13]–[15], bone morphogenetic protein family (BMP) [16], [17], and members of the transforming growth factor-β family (TGFβ) [17]–[19] have been considered as key regulators of this process. The Wnt/β-catenin (canonical) signaling pathway represents one of the key mechanisms controlling cell-fate decisions both during embryonic development and in adult tissues (reviewed in [20]). Therefore, it is not surprising that the Wnt/β-catenin signaling pathway has also been implicated in the regulation of various stages of lens development [21]–[26], including lens fiber and epithelial cell differentiation [21], [26], [27]. In the absence of Wnt ligand, β-catenin is bound to the destruction complex, phosphorylated on serine-threonine residues encoded by exon 3 (N-terminal part of β-catenin), and thus targeting the protein for proteasomal degradation. Upon binding of Wnt ligand to the Frizzled/LRP receptor complex, the destruction complex is shifted to the cell membrane and disintegrated. As a result, β-catenin accumulates in the cytoplasm and enters the nucleus, where it acts as a transcriptional co-activator and in cooperation with transcription factors of the TCF/LEF family initiates transcription of the Wnt/β-catenin target genes. β-catenin has a dual role in the cell: besides its critical role as a transcriptional co-activator of the Wnt/β-catenin signaling pathway, it also functions as a structural protein on the cell membranes in cadherin-mediated cell adhesion at adherens junctions [28]. Both of these β-catenin functions, transcriptional and cytoskeletal, have been shown to play a critical role in proper lens development and differentiation [21]–[26]. In early stages of the lens development, Wnt/β-catenin signaling has an important role in patterning the surface ectoderm. It has to be inhibited in the region of periocular ectoderm, where the lens develops, and β-catenin stabilization at E9.5 in this region results in suppression of the lens fate [23], [24]. On the contrary, active Wnt/β-catenin signaling is required in the surrounding head ectoderm. Inhibition of Wnt/β-catenin signaling at E9.5 results in formation of ectopic lenses and in defective lens cell adhesion, but has no impact on lens fate marker acquisition [22], [25]. In later stages of lens development and especially during differentiation of lens cells, the requirement for Wnt/β-catenin signaling was demonstrated in mice with null mutation of *Lrp6* that exhibit incompletely formed lens epithelium [26]. Conditional deletion of β-catenin in the whole lens, or only in differentiating fiber cells at E12.5, showed that β-catenin was required in the lens epithelium and during early fiber differentiation [21]. Loss of β-catenin in the whole lens resulted in the loss of lens epithelial marker expression (E-cadherin, Pax6) and abnormal expression of differentiating fiber cell markers (c-Maf, p57^Kip2^) in the lens epithelium, epithelial cell cycle arrest at G1-S, and premature cell cycle exit [21]. Fiber cell differentiation was also affected, with poor cell elongation, decreased β-crystallin expression, and improper formation of adhesion junctions in differentiating fiber cells [21]. β-catenin stabilization, using the Cre-loxP system in the whole lens from E12.5 onward, resulted in the ocular phenotype comprising increased progression of epithelial cells through the cell cycle and maintenance of epithelial phenotype in the fiber compartment [27]. In contrast, mice in which β-catenin stabilization was induced exclusively in lens fiber cells by embryonic day 12.5 lacked the ocular phenotype [27]. Here we show, however, that ectopic activation of Wnt/β-catenin signaling in lens fiber cells using a transgenic mouse model results in aberrant lens fiber cell differentiation and cataract formation.

## Materials and Methods

### Ethics Statement

Housing of animals and *in vivo* experiments were performed after approval by the Animal Care Committee of the Institute of Molecular Genetics (study ID#174/2010) and in compliance with national and institutional guidelines (ID#12135/2010-17210).

### Generation and Screening of Transgenic Mice

To generate αA-CLEF construct the fragment containing the coding sequence for the human C-terminal part of β-catenin (aa 696–781), followed by the mouse Lef1 coding sequence and HA-tag, was liberated from the pCMV-catCLEF1 vector [29]. This fragment was inserted into the pACP3 vector containing the αA-crystallin promoter, the SV40 small T antigen intron and polyadenylation sequence. To generate the delβ-CAT construct the fragment containing the coding sequence for HA-tagged N-terminally truncated human β-catenin (aa 91–781) was inserted into pACP3. The linearized transgenic inserts were injected into the male pronuclei of fertilized FVB/N oocytes by the Genetic Engineering Facility of National Eye Institute. αACLEF transgenic mice were further maintained on the C57Bl/6 background and identified by PCR analysis of DNA obtained by tail biopsy with the following primers: 5′ GAGGGCTGGAACGCTAGCTCA 3′, and 5′ GTCCATACCCAAGGCCTCCTG 3′. Transgenic delβ-CAT mice were identified by PCR analysis of DNA obtained by tail biopsy using primers derived from the SV40 small T intron.

### Tissue Collections and Histology

Mouse embryos were staged by designation the noon of the day when the vaginal plug was observed as embryonic day 0.5 (E0.5). Embryos of the required developmental stage were harvested in cold PBS, fixed in 4% PFA/PBS (w/v) for 2–4 h on ice. Tissue was cryoprotected by overnight incubation in 30% sucrose (w/w) at 4°C, embedded and frozen in OCT (Tissue-Tek, Sakura Finetek). Horizontal cryosections of 12 µm were prepared, stored at −20°C and used up to two weeks.

For paraffin sections, E16.5 embryos were fixed in 8% PFA/PBS (w/v) overnight on ice, processed and embedded in paraffin and sectioned 7 µm, stored at 4°C and used up to one month.

### Immunofluorescence

The cryosections were air-dried for at least 30 min, washed with PBS, and permeabilized with PBS/0,1% Triton X-100 (PBT) for 15 min prior to blocking. For some antibodies the antigen retrieval was performed: slides were immersed in 10 mM citrate buffer (pH 6.0) in coplin jars and boiled for 5–15 min, then let cool down to room temperature. The paraffin sections were air-dried for at least 30 min, deparaffinized and rehydrated. Epitope retrieval was performed in 10 mM citrate buffer (pH 6.0) for 15 min in pressure cooker. All sections were blocked for 30 min in 10% BSA/PBT (w/v), incubated overnight with primary antibody at 4°C (diluted in 1% BSA/PBT), washed three times with PBS, incubated 45 min at room temperature with secondary antibody, washed three times with PBS, incubated 15 min with DAPI/PBS, washed with PBS and mounted into Mowiol 4–88. Primary antibodies used included: Pax6 (Covance, PRB-278P), Prox1 (Chemicon, AB5475), Sox1 (Santa Cruz, sc-17317), c-Maf (Bethyl Laboratories, BL662), Foxe3 (Peter Carlsson), cyclin D1 (Santa Cruz, sc-450), cyclin D2 (Santa Cruz, sc-452), p27^Kip1^ (Santa Cruz, sc- 528), p57^Kip2^ (Santa Cruz, sc-1039), α-Smooth Muscle Actin (Sigma, A5228), N-cadherin (DSHB, MNCD2-c), Z0-1 (DSHB, R26.4C), Cleaved Caspase-3 (Cell Signaling, 9664), β-crystallin (Samuel Ziegler), γ-crystallin (Hisato Kondoh). Secondary antibodies used were the following: Alexa-488- or 594-conjugated donkey anti-rabbit, anti-mouse, anti-goat, or anti-rat IgG (Molecular Probes). Standard histological staining of cryosections by hematoxylin and eosin (H&E) was also performed. At least three different embryos from at least two different litters were analyzed with each staining.

### Total RNA Isolation, cDNA Synthesis and RT-PCR

Total RNA was isolated from dissected eyes or lenses with Trizol® Reagent (Invitrogen), contaminating DNA was eliminated by DNAse I digestion and RNA was repurified with RNeasy Micro kit (Qiagen). Random-primed cDNA was generated from 500 ng of total RNA using SuperScript VILO cDNA Synthesis kit (Invitrogen). Primers 5′ GTGAAGGAACCTTACTTCTGTGGTG 3′, and 5′ GTCCTTGGGGTCTTCTACCTTTCTC 3′, derived from the SV40 small T intron, were used for RT-PCR detection of transgenic CLEF mRNA. Total RNA was isolated from two lenses of one E16.5 embryo, or newborn with Trizol® Reagent. At least three different E16.5 embryos were used for total RNA isolation. Random-primed cDNA was generated from 200 ng of total RNA using SuperScript VILO cDNA Synthesis kit (Invitrogen) and two independent synthesis of cDNA were performed from one total RNA sample.

### Quantitative RT-PCR (qRT-PCR)

qRT-PCR were run in the LightCycler® 480 Instrument (Roche) using LightCycler® 480 DNA SYBR Green I Master (Roche) according to the standard manufacturer’s protocol; typically, 5 µl reaction mixture was used. PCR reactions were performed in triplicate for each primer set of primers, with three different cDNA (biological triplicate). Control reactions (containing corresponding aliquots from cDNA synthesis reactions that were performed without reverse transcriptase; minus RT controls) were run in parallel. Crossing point (Cp) values were calculated by LightCycler® 480 Software (Roche) using the second-derivate maximum algorithm. The average Cp values of all biological and technical replicates were normalized by Cp values of housekeeping genes. Statistical significance of the change in mRNA expression was calculated by a two-tailed Student t-test in Microsoft Excel. Finally, the change in mRNA expression of αA-CLEF lenses is presented as the ratio αA-CLEF/wild-type in log with base 2 scale. Primer sequences are listed in Table S1.

### Western Blot Analysis

Lenses were dissected from 8–10-month-old wild-type and αA-CLEF mice, mechanically homogenized in total lens protein lysis buffer [30] or in soluble lens protein lysis buffer [31], sonicated (4×10 sec, 30%), centrifuged at 14 000× g/4°C/8 min, the supernatant was collected, and the protein concentration was determined using BCA™ Protein Assay Kit and *NanoDrop ND1000.* One µg (for β-crystallin detection) and 15 µg (for α- and γ-crystallin detection) of protein were denatured in 2× SDS, boiled and subsequently separated by SDS-PAGE, and transferred onto nitrocellulose membranes. Membranes were blocked with 5% non-fat milk/PBT for 2 h at RT, incubated with primary antibody (anti- α-crystallin and β-crystallin, Samuel Ziegler; γ-crystallin, Hisato Kondoh; HRP-conjugated anti-β-actin, Sigma; anti- β-catenin, Sigma) diluted in 5% milk/PBT at 4°C overnight, washed 3 times in PBT and incubated with HRP-conjugated species-specific secondary antibody for 2 h (HRP-conjugated anti-rabbit swine secondary antibody, Dakocytomation), and washed 3 times in PBT. The signal was detected by enhanced chemi-luminescence (ECL) kit (Amersham Bioscience). To compare protein levels, quantification of the blots was performed using Aida Image Analyser Software. Around 15 lenses dissected from E16.5 wild-type or αA-CLEF embryos were pooled and homogenized in 10 µl of total lens protein lysis buffer; 50 µg of protein was loaded on SDS-PAGE. CLEF transgenic protein was detected with primary anti-HA-High Affinity rat primary antibody and subsequently with HRP-conjugated anti-rat goat secondary antibody (Pierce).

## Results

### β-catenin Stabilization in Lens Fiber Cells Results in Cataract Development in Adulthood

**Figure 1:**
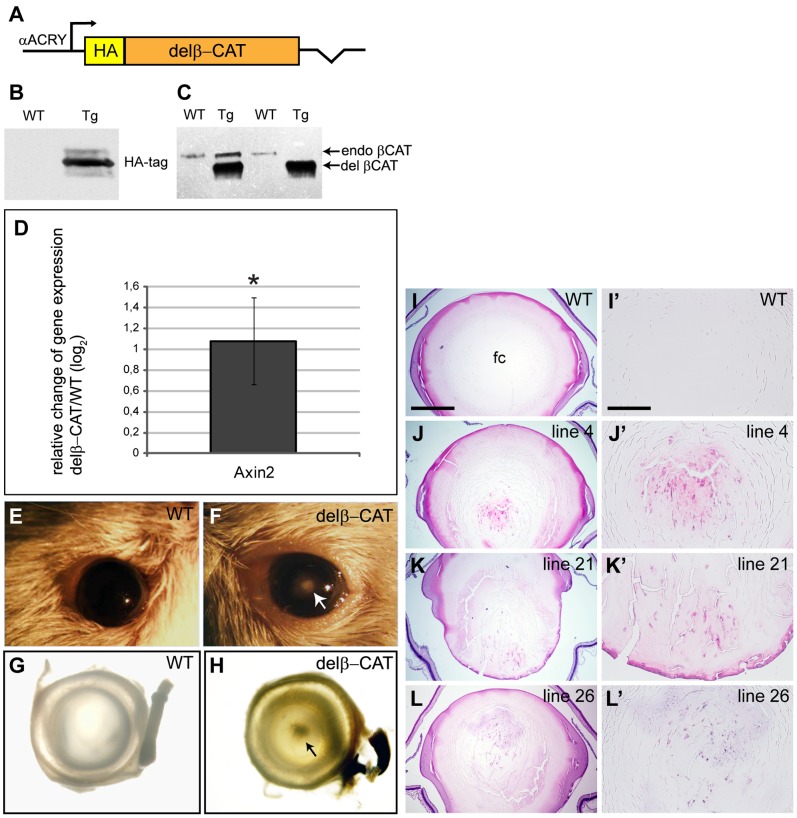
β-catenin stabilization in lens fiber cells results in cataract. (A) Schematic diagram of delβ-CAT transgenic construct. (B) delβ-CAT transgenic protein is detected in adult mutant lenses (Tg) with anti-Ha-tag antibody. (C) N-terminally deleted β-catenin is detected in transgenic lenses (Tg) with anti-β-catenin antibody. Note that the delβ-cat protein is expressed in higher amount than endogenous β-catenin. (D) qRT-PCR demonstrates upregulated mRNA expression of Axin2 in newborn delβ-CAT lenses (*p<0.05). Ocular phenotype of adult wild-type (E, G) and delβ-CAT (F, H) mice, adult transgenic mice develop cataract, indicated with arrows (F, H). (I-L) Histological sections of adult wild-type (I) and transgenic (J, K, L) eyes and detail view of wild-type (I′) and delβ-cat (J′, K′, L§) lens fiber cell compartment (fc). Scale bars indicate (I, J, K, L) 500 µm and (I′, J′, K′, L′) 200 µm.

Activation by Wnt/β-catenin signaling leads to inhibition of the GSK3 kinase that causes phosphorylation of β-catenin’s N terminus and targets its cytoplasmic pool for ubiquitin-mediated degradation. N-terminal truncation of β-catenin leading to increased levels of β-catenin was therefore previously used to activate Wnt/β-catenin signaling in the mouse skin [32]. To investigate the effect of ectopic activation of Wnt/β-catenin signaling in eye lens we have initially generated a transgenic mouse, referred to as delβ-CAT mouse, expressing N-terminally truncated β-catenin (aa 91–781) in lens fiber cells under the control of αA-crystallin promoter (−342/+49), (Fig. 1A). Presence of transgenic delβ-CAT protein in adult mutant lenses was detected by anti-HA tag antibody on western blot (Fig. 1B). N-terminally truncated β-catenin was detected with anti-β-catenin antibody confirming that the delβ-CAT protein level was significantly higher than the expression of endogenous β-catenin in adult delβ-CAT lenses (Fig. 1C). Upregulated expression of Axin2, known Wnt/β-catenin signaling target gene, in newborn lenses indicated the Wnt/β-catenin signaling activation in delβ-CAT lenses. Several independent founders of delβ-CAT mice developed cataract starting from 3 weeks of age (Fig. 1E-L’). These results suggested that expression of stabilized β-catenin is not compatible with the normal development and homeostasis of the lens. However, as β-catenin stabilization can result either in transcriptional activation of Wnt/β-catenin target genes or in accumulation of β-catenin on the cell membranes [28], it was not obvious which of the β-catenin functions was responsible for the cataract development in adult delβ-CAT mice. In order to eliminate the impact of a cytoskeletal function of β-catenin on cataract formation, we generated another transgenic mouse where only the transcriptional function of β-catenin was manipulated to specifically achieve activation of Wnt/β-catenin signaling (see below).

### Wnt/β-catenin Signaling Activation in Lens Fiber Cells Results in Cataract Development and Disrupted Lens Morphology

To activate Wnt/β-catenin signaling in lens fiber cells from the stage E12.0 onward, the transgenic construct designated αA-CLEF was generated. Constitutive activation of Wnt/β-catenin signaling was achieved by expression of transgenic protein CLEF containing the C-terminal activation domain of β-catenin fused to the amino terminus of the full-length protein Lef1. An HA-tag was fused to the carboxy terminus of Lef1 to simplify detection of the transgene expression. An identical fusion protein has previously been used to mimic activation of Wnt/β-catenin signaling [29], [33]. Since we have used the CLEF fusion protein to activate Wnt/β-catenin signaling, we can exclude the possibility that the phenotypes observed here are due to the cytoskeletal function of β-catenin.

**Figure 2:**
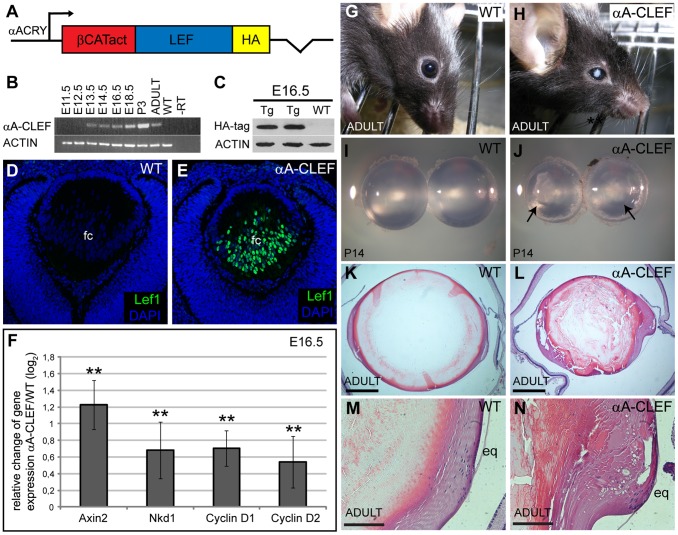
Ectopic activation of Wnt/β-catenin signaling in lens fiber cells results in microphthalmia and cataract. (A) Schematic diagram of αA-CLEF transgenic construct. αA-crystallin promoter (αACRY) drives expression of the fusion protein containing the C-terminal transactivation domain of human β-catenin fused to the amino terminus of full-length mouse Lef1 and HA-tag. (B) RT-PCR analysis of the onset of transgenic mRNA. CLEF mRNA is fully detected in mutant eyes from E13.5 until onward. (C) CLEF transgenic protein is detected in mutant lenses (Tg) at E16.5 with anti-HA-tag antibody. (D, E) CLEF transgenic protein is detected with anti-Lef1 antibody in the central part of the fiber cell compartment of mutant lenses at E13.5. (F) qRT-PCR demonstrates relative change of gene expression in E16.5 αA-CLEF transgenic lenses. Expression of known Wnt/β-catenin signaling target genes – Axin2, Nkd1, Cyclin D1 and Cyclin D2 is upregulated in E16.5 αA-CLEF lenses compared to wild-type, (**p<0.01). (G-N) Ocular phenotype of wild-type (G, I, K, M) and αA-CLEF (H, J, L, N) mice. (G, H). Adult transgenic mice develop microphthalmia and cataract. (I, J) Dissected lenses from P14 transgenic mouse are already smaller (J) compared to wild-type lenses (I) and visible cataract is already present in P14 transgenic lenses (J). (K-N) Histological sections of adult wild-type (K) and transgenic (M) eyes and detail view of wild-type (M) and αA-CLEF (N) lens equatorial region. Disorganized fiber cell nuclei and vacuoles are present in the equatorial region of transgenic lens (N). Scale bars indicate (D, E) 50 µm, (K, L) 400 µm, and (M, N) 100 µm. Abbreviations: fc, fiber cell compartment; eq, equatorial region.

Fiber cell-specific expression of the CLEF fusion protein was achieved by αA-crystallin promoter (−342/+49) [34], (Fig. 2A). Three independent founders harboring the transgene were generated. All three founders developed cataracts in adulthood (data not shown) and their lenses appeared to have disrupted morphology (Fig. S1). Two independent transgenic lines (MB06-05 and MB06-12) were established and further examined. As animals of both lines manifested the same ocular phenotype, the MB06-5 line was used for all of the subsequent analyses and was further referred to as αA-CLEF. αA-CLEF mice were maintained and analyzed as heterozygotes on the C57Bl/6 background. In order to establish the onset of CLEF mRNA expression in the lens, a sensitive RT-PCR was initially performed. Since αA-crystallin promoter is active from embryonic day 12.5 [34], we examined the transgene expression in eyes starting from developmental stage E11.5. Only weak expression of the transgene was detected at E12.5, whereas from E13.5 onward the CLEF mRNA expression was easily detected in eyes of αA-CLEF transgenics (Fig. 2B). The presence of the CLEF fusion protein in lenses of αA-CLEF transgenic mice was confirmed at E16.5 using anti-HA-tag antibody (Fig. 2C). In order to detect the CLEF protein within the embryonic lens, anti-Lef1 antibody was used for immunofluorescent detection. Under the conditions used, Lef1 expression was not detected in wild-type lenses (Fig. 2D). As expected, given the known selectivity of αA-crystallin promoter, the CLEF protein was clearly present in the nuclei of fiber cells already in transitional zone and in the central fiber cell compartment of αA-CLEF transgenic mice (Fig. 2E). Since endogenous mouse Lef1 mRNA was not upregulated in αA-CLEF transgenics (unpublished data), the signal obtained by immunofluorescence represents the exogenous CLEF protein. Wnt/β-catenin signaling activation was confirmed by upregulated mRNA expression of known Wnt/β-catenin target genes – Axin2, Nkd1, cyclin D1 and cyclin D2 in E16.5 αA-CLEF lenses (Fig. 2F). Adult transgenic αA-CLEF mice developed cataract and microphthalmia (Fig. 2G, H). Cataract was present already in lenses of two-week-old (P14) transgenic αA-CLEF mice and lenses were smaller compared to lenses from wild-type littermates at that stage (Fig. 2 I, J). Histological examination of eyes from the F0 adult αA-CLEF animals confirmed smaller size of the whole eye and exhibited a number of morphological alterations. Detailed view of the transitional region of mutant lens revealed random distribution of fiber cell nuclei throughout the fiber cell region, and some regions of the lens contained vacuoles (Fig. 2 K-N). Additional histological analysis of mutant lenses of subsequent generations confirmed that the ocular phenotype was transmitted (data not shown).

### αA-CLEF Lenses are Characterized by Decreased Levels of γ-crystallins

**Figure 3:**
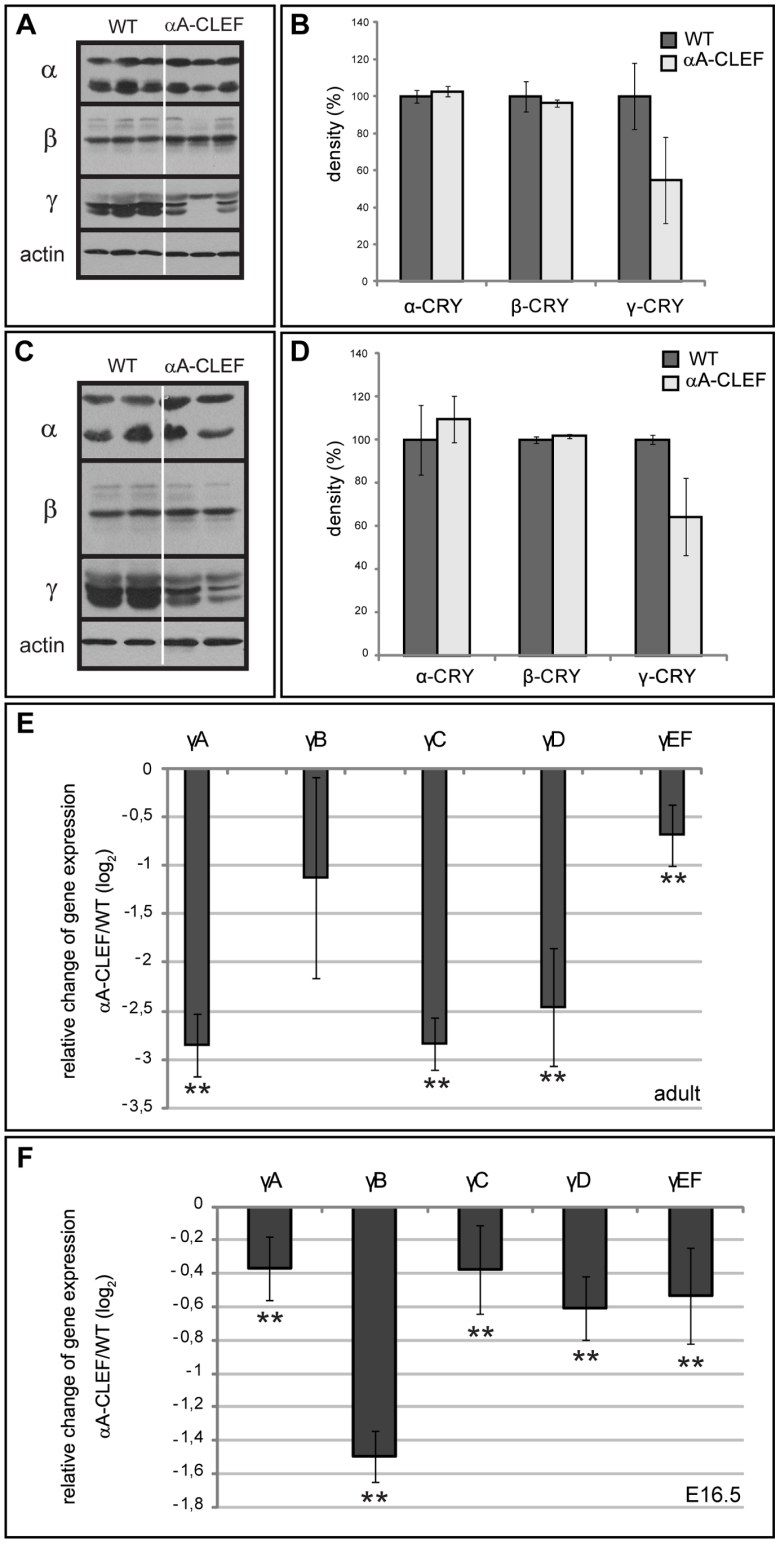
Downregulation of γ-crystallin protein and mRNA in adult αA-CLEF lenses. (A, C) Western blot analysis shows less γ-crystallin in total (A) and soluble (C) protein extract of adult αA-CLEF lenses compared to wild-type. (B, D) Quantification of band density of total (B) and soluble (D) lens protein extract western blot analysis. (E) Quantitative RT-PCR expression analysis of γ-crystallins in adult wild-type and αA-CLEF lenses. mRNA expression of γA-, γC-, γD- and γEF-crystallin is significantly lower in adult αA-CLEF lenses. (F) Quantitative RT-PCR expression analysis of γ-crystallins in E16.5 wild-type and αA-CLEF lenses. mRNA expression of γA-, γB-, γC-, γD- and γEF-crystallin is significantly lower already in E16.5 αA-CLEF lenses (**p<0.01).

It is well established that β- and γ-crystallins are present in very high concentrations in the lens, and in altered conditions can precipitate within the lens cells to form the insoluble fraction, thus contributing to cataract formation (reviewed in [35]). We therefore examined whether the crystallin protein level was not changed in adult cataractous αA-CLEF lenses. Total and soluble protein extracts were prepared from lenses of 8–10-month-old mice, when the cataract was obvious in αA-CLEF lenses. At least two independent samples were prepared from wild-type lenses and from αA-CLEF lenses, and α-, β- and γ-crystallin proteins were detected by specific antibodies on western blot. Less γ-crystallin was detected in lenses of adult αA-CLEF mice in the total lens protein extract (Fig. 3A, B), as well as in the soluble lens protein extract (Fig. 3C, D). In order to distinguish whether lower levels of γ-crystallin protein in adult αA-CLEF lenses were caused by aggregation (and therefore impossible to be detected either in total or in soluble protein fraction), or by a lower level of transcription of γ-crystallin coding genes, qRT-PCR was performed. Quantitative RT-PCR analysis revealed that the expression of γA-, γC-, γD-, and γE/F-crystallin mRNA was significantly decreased in adult αA-CLEF lenses in comparison to wild-type lenses of equal age (Fig. 3E). As the presence of CLEF mRNA and CLEF fusion protein was confirmed in E13.5 and E16.5 αA-CLEF lenses, respectively, the expression of crystallin genes was examined at these stages of lens development. First, the expression of β- and γ-crystallin in developing lens at E13.5 and at E16.5 in wild-type and αA-CLEF lenses was analyzed by imunofluorescence. No apparent difference was observed in the expression levels of β- and γ-crystallin in E13.5 or in E16.5 αA-CLEF lenses compared to wild-type lenses (Fig. S2). However, qRT-PCR analysis showed that the expression of γ-crystallin mRNA was significantly decreased in αA-CLEF lenses in comparison to wild-type lenses already at E16.5 (Fig. 3F). In summary, we provide evidence of decreased levels of γ-crystallins in αA-CLEF lenses beginning at E16.5 and persisting until adulthood.

### Fiber Cell Junctions are not Affected in Embryonic αA-CLEF Lenses

In order to exclude the impact of αA-CLEF transgene on normal fiber cell junctions, localization of tight junction protein zona occludens-1 (ZO-1) and localization of adherens junction molecule N-cadherin was examined at E13.5 and E16.5 in wild-type and αA-CLEF lenses. ZO-1 has been previously shown to localize to the apical membrane of epithelial cells (epithelium-fiber cell interface) within the developing lens [36]. Strong expression of ZO-1 was observed on the apical aspect of the lens epithelial cell layer in wild-type as well as in αA-CLEF lenses at E13.5 and E16.5 (Fig. S3A-D). N-cadherin has been shown to be expressed in both lens epithelial cells and fiber cells during lens differentiation [37], the same expression pattern was observed in αA-CLEF lenses at E13.5 and E16.5 (Fig. S3E-H). Because no significant difference was observed in the localization of ZO-1 and N-cadherin in αA-CLEF lenses, we assumed that fiber cell junctions were not affected in αA-CLEF lenses.

### Epithelial-mesenchymal Transition and Apoptosis are not Induced in Embryonic αA-CLEF Lenses

As active Wnt/β-catenin signaling has been asociated with epithelial-mesenchymal transition (EMT), presence of α-SMA (α-smooth muscle actin), marker of EMT, was examined in E13.5 and E16.5 αA-CLEF lenses. α-SMA is normaly expressed within the overlying iris tissue and muscles of eyelids. We did not observe any positive staining for α-SMA in E13.5 or in E16.5 αA-CLEF lenses (Fig. S4A-D-D) indicating the absence of EMT during the process of abnormal lens differentiation in αA-CLEF transgenic mice.

To determine whether Wnt/β-catenin signaling activation leads to apoptosis in αA-CLEF lens fiber cells, we examined the levels of cleaved caspase 3 (marker of apoptotic cells)-positive cells by immunofluorescence. No cleaved caspase 3-positive nuclei of fiber cells were detected in E13.5 and E16.5 αA-CLEF lenses, similary to E13.5 and E16.5 wild-type lenses (Fig. S4E-H).

### Expression of Transcriptional Regulators in Embryonic Lenses of αA-CLEF Transgenic Mice

**Figure 4:**
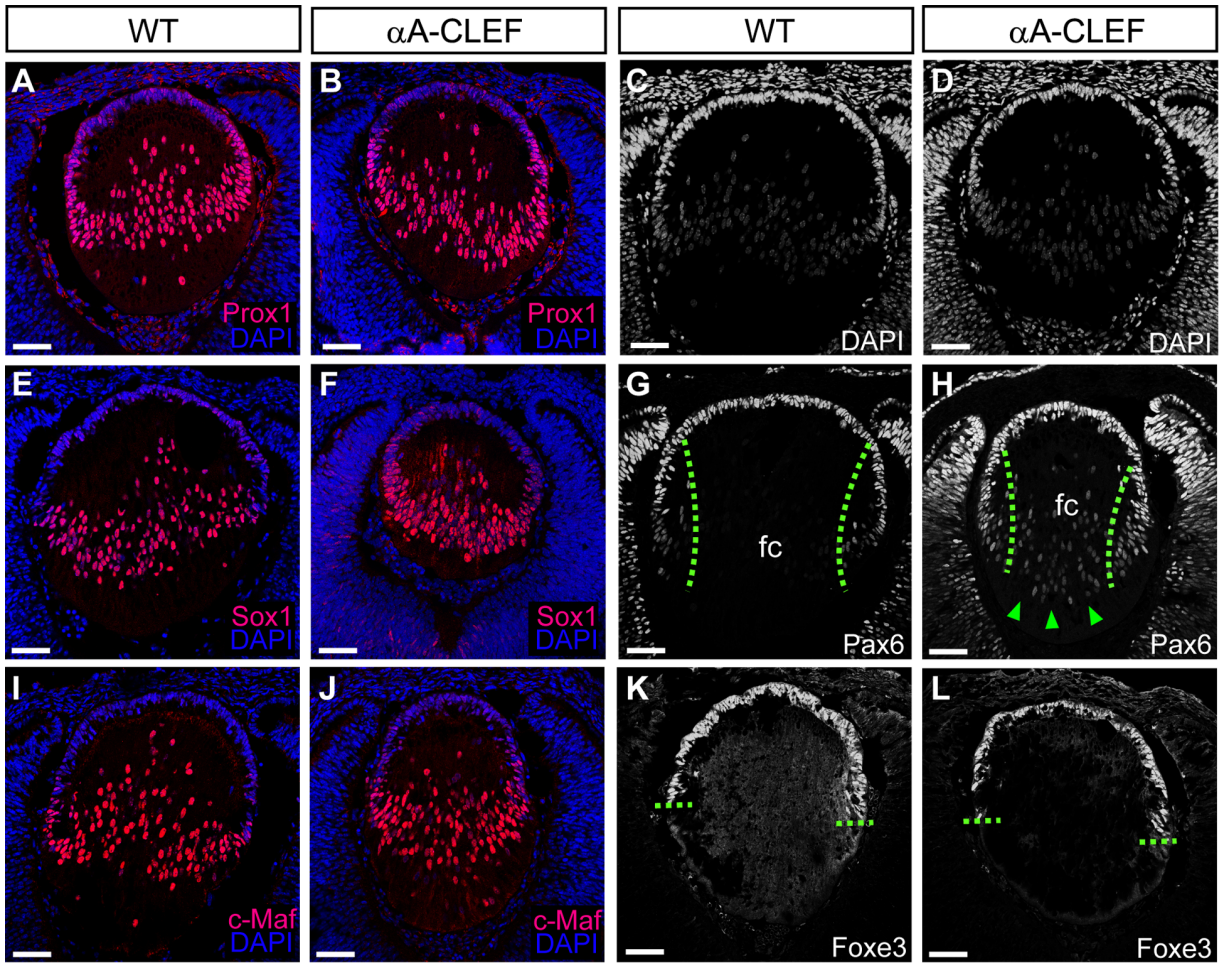
Developmental expression of transcriptional regulators in αA-CLEF mice at E13.5. Cryosections of wild-type (A, C, E, G, I, K) and αA-CLEF (B, D, F, H, J) embryos stained for DAPI (C, D), for early differentiation marker Prox1 (A, B), Sox1 (E, F), for marker of fiber cell differentiation c-Maf (I, J), for epithelial cell marker Pax6 (G, H) and its target Foxe3 (K, L), with parallel DAPI counterstaining. There is no difference in the distribution of fiber cell nuclei or in the expression of Foxe3, Sox1, Prox1 and c-Maf. (H) Pax6 expression persists in the fiber cell compartment of the lens of αA-CLEF embryo (indicated with green arrowheads), whereas its expression is lost in the equatorial region of lens during fiber cell differentiation in wild-type embryos (G). Scale bars indicate 50 µm. Abbreviations: fc, fiber cell compartment; eq, equatorial region.

To address the sequence of events leading to cataract formation in adulthood, we examined the localization of fiber cell nuclei and expression of key lens regulatory proteins in developing embryonic lenses. We have chosen E13.5 as the starting point since at this developmental stage CLEF mRNA begins to be highly expressed. Despite abnormal fiber cell nuclei localization in adult αA-CLEF lenses, DAPI staining showed that the distribution of fiber cell nuclei was unaltered at E13.5 (Fig. 4C, D). Transcription factors c-Maf, Sox1 and Prox1 are involved in proper fiber cell differentiation and crystallin expression [4], [5], [38] and at E13.5 are predominantly expressed in fiber cell nuclei of wild-type lenses [4], [5], [38]. Similar expression was observed in αA-CLEF lenses (Fig. 4A, B, E, F, I, J). In contrast, Pax6 expression remains high in the anterior lens epithelium and lens equator, and weakens as the cells undergo differentiation at the transitional zone [39]. Expression of Foxe3, a known target gene of Pax6, specifies lens epithelial cells [40], [41]. The expression of Foxe3 in αA-CLEF lenses appeared to be unaltered (Fig. 4K, L), whereas Pax6 expression was stronger in the nuclei of fiber cells in the fiber cell compartment compared to wild-type (Fig. 4G, H).

**Figure 5:**
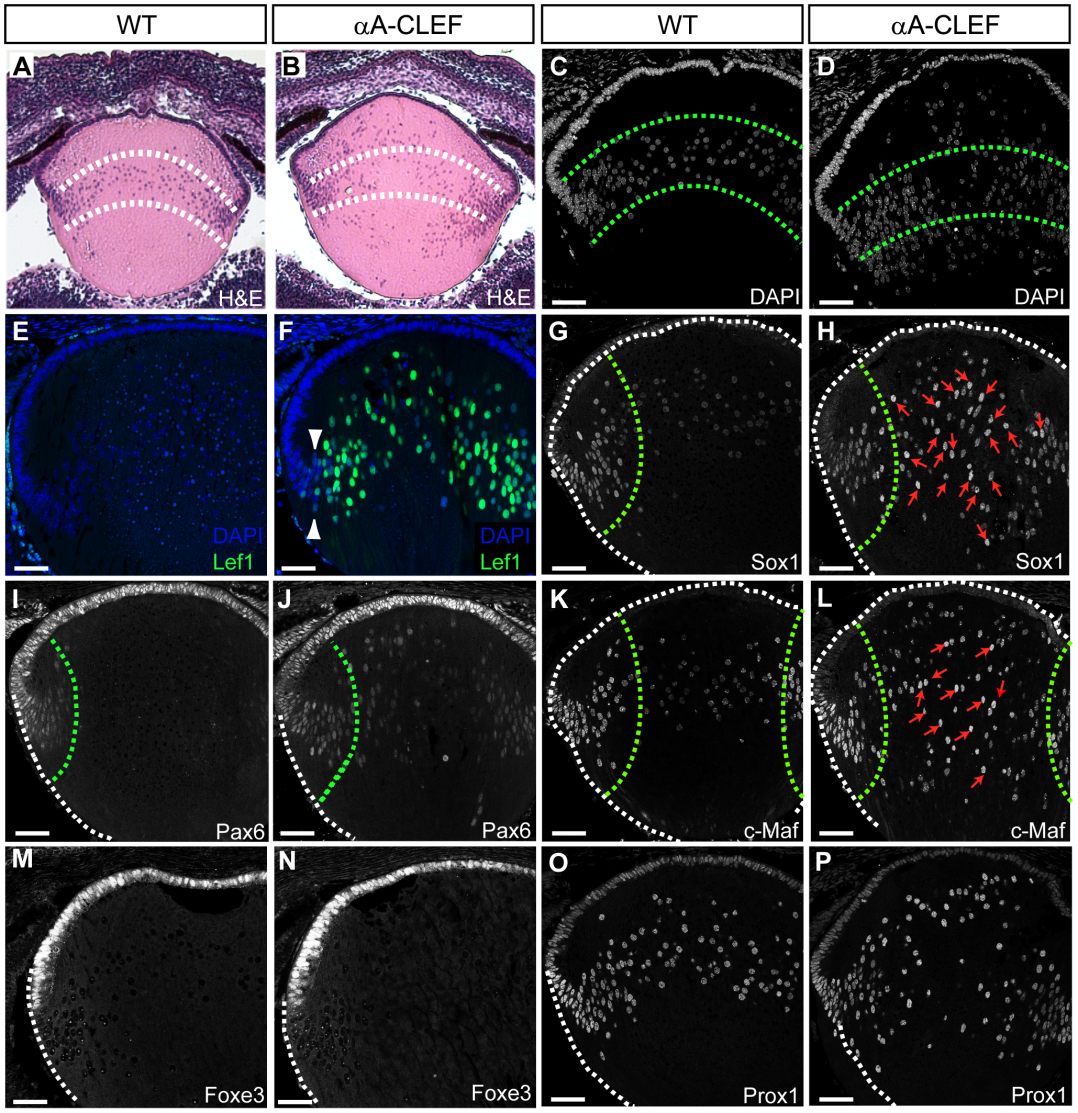
Ectopic Wnt/β-catenin signaling activation affects fiber cell nuclei localization and expression of lens regulatory proteins. Cryosections of E16.5 wild-type (A, C, E, G, I, K, O) and αA-CLEF (B, D, F, H, J, L, N, P) embryos stained with hematoxylin and eosin (A, B), DAPI (C, D), for lens epithelial cell marker Pax6 (I, J), its target Foxe3 (M, N), for early differentiation marker Prox1 (O, P) and for fiber cell differentiation markers Sox1 (G, H) and c-Maf (K, L). (E, F) CLEF transgenic protein is detected with anti-Lef1 antibody nuclei of fiber cells from transitional zone to fiber cell compartment. Fiber–cell-nuclei are detected throughout the fiber cell compartment in αA-CLEF lenses (B, D), and the expression of Pax6 (F), Sox1 (H) and c-Maf (L) is stronger in the fiber cell compartment of αA-CLEF lenses (indicated with red arrows) compared to wild-type (E, G, K). Scale bars indicate 50 µm.

To further explore changes in the lens fiber cell differentiation, we investigated the expression of fiber cell differentiation regulators and the position of fiber cell nuclei in a later stage of lens development, at E16.5. In lenses of E16.5-old controls, the nuclei of the equatorial transitional zone, where lens epithelial cells undergo differentiation, were organized in a characteristic bow pattern (Fig. 5A, C). By contrast, in αA-CLEF lenses, nuclei of differentiating fiber cells were disorganized in the entire lens fiber region (Fig. 5B, D). The same disorganization of fiber cell nuclei showed staining of Prox1-positive nuclei; moreover, Prox1 reactivity appeared more intensive in the nuclei in the central part of the fiber compartment of αA-CLEF lenses compared to wild-type lenses (Fig. 5O, P). Based on immunostaining of CLEF transgenic protein (Fig. 5E, F) it is apparent that the fiber cells from the transitional zone towards the center of the lens were affected by the expression of transgene at E16.5 αA-CLEF lenses. At E16.5 the expression of transcription factors regulating the differentiation of fiber cells was almost lost or weaker in the central part of the fiber cell region of wild-type lenses (Fig. 5I, G, K). However, in αA-CLEF lenses, Pax6, Sox1 and c-Maf strongly positive nuclei were detected in the fiber cell region of the lens (Fig. 5J, H, L). In contrast, the expression of Foxe3 in E16.5 αA-CLEF lenses appeared to be unaltered (Fig. 5M, N). Combined, our data indicate abnormal expression of some key transcription factors during lens fiber cell differentiation in αA-CLEF transgenic mice.

### Activated Wnt/β-catenin Signaling in Lens Fiber Cells Affects the Cell Cycle Exit of Differentiating Fiber Cells in αA-CLEF Lenses

**Figure 6:**
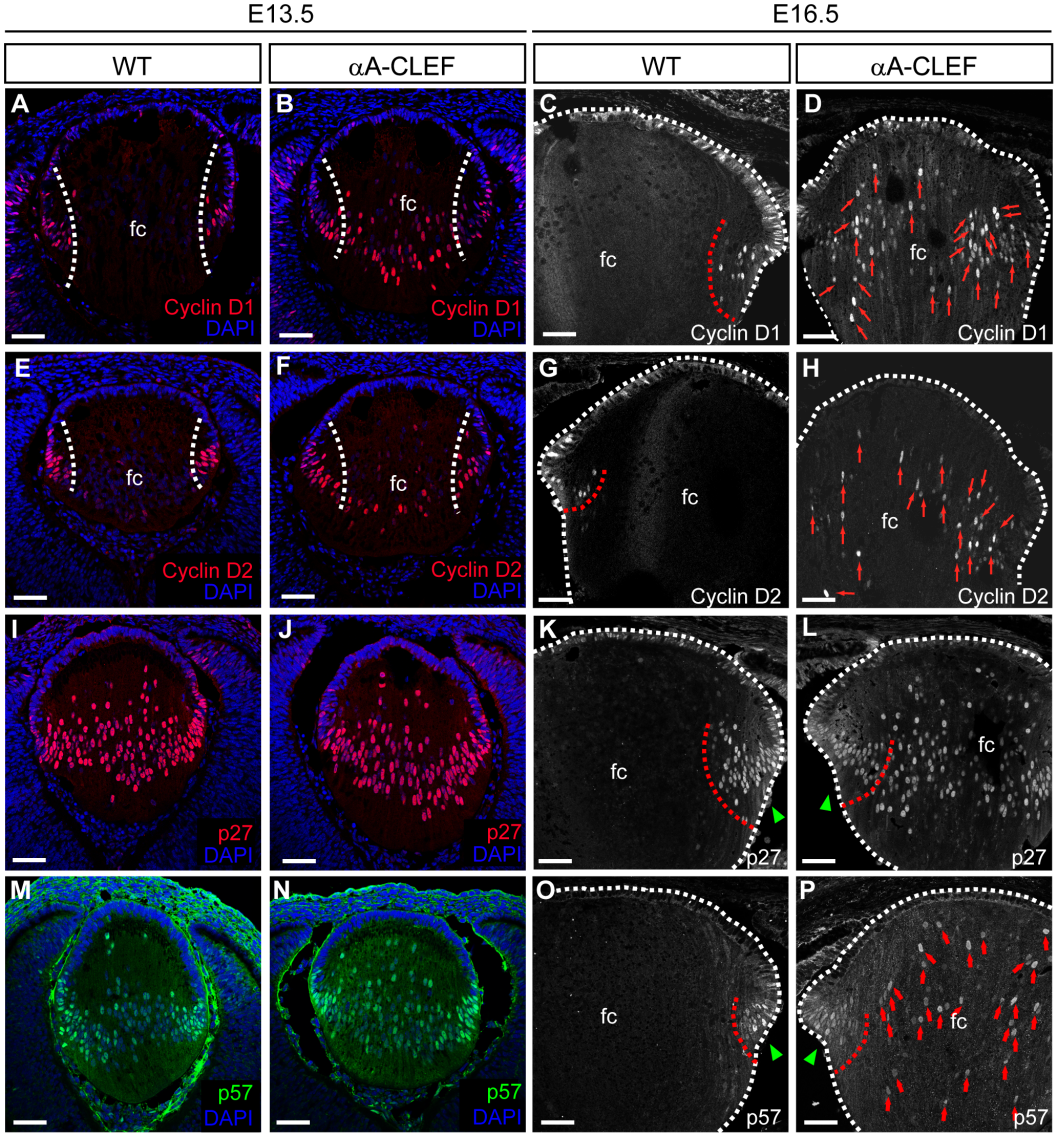
Expression of cell cycle markers persists in the fiber cell compartment of αA-CLEF lenses. (A) Cyclin D1 and (E) cyclin D2 expression is detected mainly in the equatorial region and transitional zone of wild-type lenses at E13.5. However, in αA-CLEF lenses, cyclin D1 (B) and cyclin D2 (F) reactivity is detected in the fiber cell compartment. (J) p27^Kip1^ and (N) p57^Kip2^ expression is unaltered in E13.5 αA-CLEF lenses compared to wild-type lenses (I, M). At E16.5, (D) cyclin D1, (H) cyclin D2, (I) p27^Kip1^ and (P) p57^Kip2^ expression is inappropriately maintained in the fiber compartment in the central part of αA-CLEF lenses compared to wild-type lenses (C, G, K, O), where the highest levels of expression are normally observed at the transitional zone (indicated with green arrowheads) at E16.5. Scale bars indicate 50 µm. Abbreviations: fc, fiber cell compartment.

Since active Wnt/β-catenin signaling is known to regulate the cell cycle [42], and as the persistence of fiber cell differentiation markers in the fiber cell compartment of transgenic αA-CLEF lenses suggested delayed differentiation of fiber cells, we examined the expression of cell cycle promoting factors cyclin D1, cyclin D2 and the expression of negative regulators of cell cycle p27^Kip1^ and p57^Kip2^ [43]. In wild-type E13.5 lenses, cyclin D1 and cyclin D2 were strongly expressed in transitional and germinative zones, and cyclin D1 was also weakly expressed in some of the lens epithelial cells (Fig. 6A, E). Virtually the same expression pattern was observed later at E16.5 (Fig. 6C, G). Cyclin-dependent kinase inhibitors p27^Kip1^ and p57^Kip2^ were strongly expressed in transitional zone and further in fiber cells in the fiber cell compartment of E13.5 wild-type lenses (Fig. 6I, M), whereas later at E16.5 the expression was limited to the transitional zone (Fig. 6K, O). Immunofluorescent analysis of E13.5 αA-CLEF lenses has shown that both cyclin D1 and cyclin D2 were abnormally expressed already at this developmental stage, as they persisted in the nuclei of fiber cells in the fiber cell compartment (Fig. 6B, F). The expression of cyclin-dependent kinase inhibitors p27^Kip1^ and p57^Kip2^, which regulate cell cycle exit, appeared unaltered at E13.5 (Fig. 6J, N). In E16.5 αA-CLEF lenses expression of cyclin D1, cyclin D2 as well as p27^Kip1^ and p57^Kip2^ persisted in the fiber compartment (Fig. 6D, H, L, P) in contrast to wild-type lenses, where the expression of these cell cycle regulators was reduced to the lens epithelium and to the transitional zone (Fig. 6C, G, K, O). Our data provide evidence of aberrant expression of cell cycle regulators in embryonic lenses of αA-CLEF transgenic mice. Combined, our observations suggest that active Wnt/β-catenin signaling in fiber cells results in a delay in the cell cycle exit and a shift of the fiber cell differentiation to the central fiber cell compartment.

## Discussion

**Figure 7:**
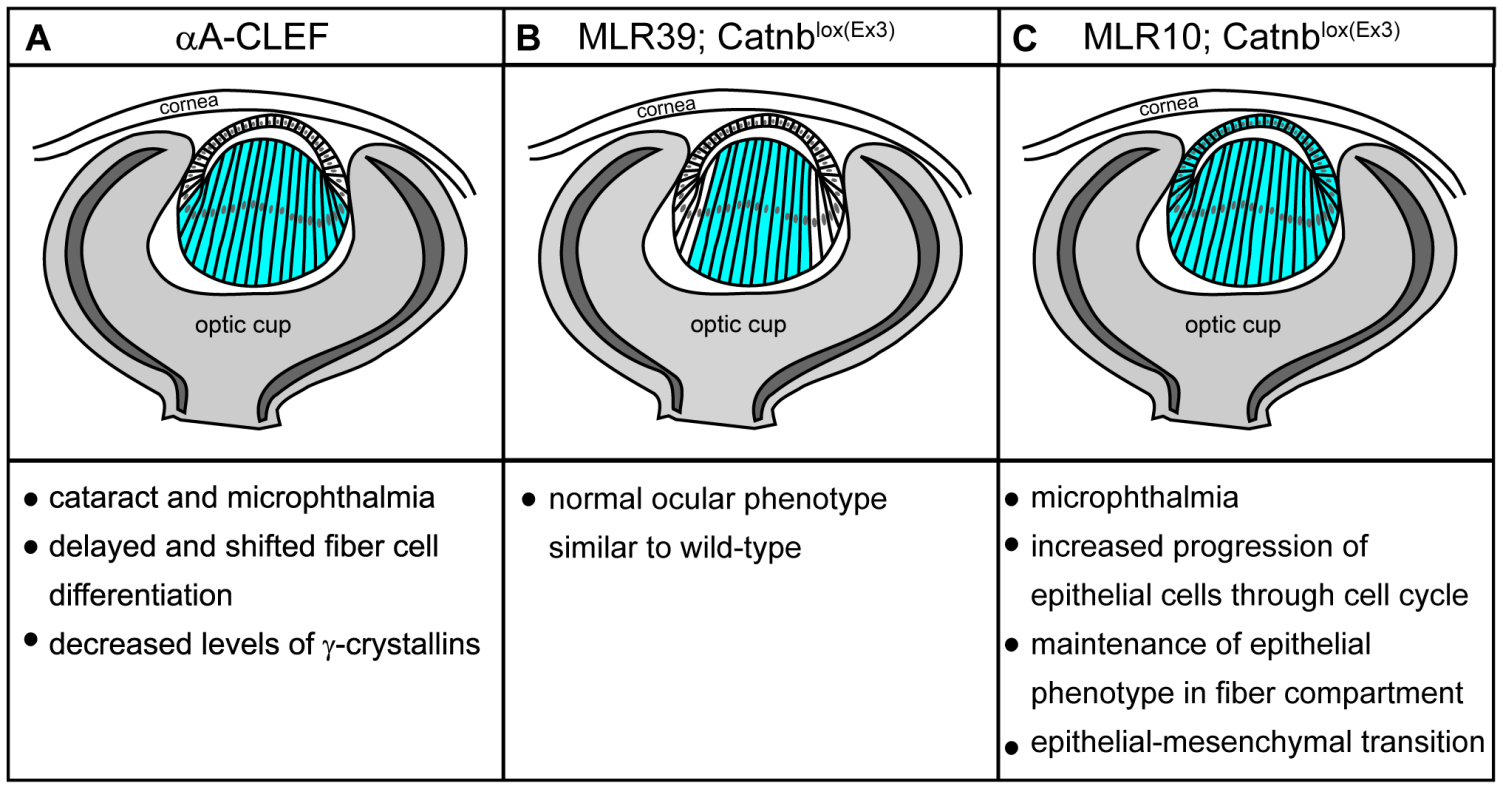
Schematic representation of the consequences of ectopic Wnt/β-catenin signaling activation, or β-catenin stabilization in a specific lens region from E12.0. (A) αA-CLEF (B) MLR39; Catnb^lox(Ex3)^ [27] (C) MLR10; Catnb^lox(Ex3)^ [27]. Picture of eye illustrates stage E12.0. Blue color represents the part of the lens where the activity of Wnt/β-catenin signaling or the stability of β-catenin was manipulated.

The Wnt/β-catenin signaling pathway controls many processes during development, including cell proliferation, cell differentiation and tissue homeostasis, and its aberrant regulation has been linked to various diseases in man (reviewed in [20]). In this study we have investigated the effect of ectopic activation of Wnt/β-catenin signaling during lens fiber cell differentiation. A previous study has shown that activation of Wnt/β-catenin signaling using stabilization of β-catenin in lens fiber cells by means of the Cre/loxP system did not manifest an obvious lens phenotype [27]. In contrast, we show here that Wnt/β-catenin activation using a transgenic approach results in a conspicuous lens phenotype, which includes aberrant fiber cell differentiation detectable as early as at E13.5 during embryogenesis, and cataract development and microphthalmia accompanied by low levels of γ-crystallin proteins and mRNA. The main results of both studies are summarized in Figure 7. The reasons for an apparent difference in phenotypes presented here and in [27] are currently unclear. It is likely, however, that they are due to the different experimental approaches used to activate the Wnt/β-catenin signaling pathway. We have observed cataract formation in transgenic mice designated delβ-CAT, which express a stabilized (non-destructible) form of β-catenin, as well as in mice expressing a fusion protein of the Lef1 transcription factor and transactivation domain of β-catenin. In both cases, the mouse αA-crystallin promoter known to be selectively expressed in lens fiber cells [34] was used to drive expression of the transgenic protein. N-terminal truncation of β-catenin and direct fusion of the transactivation domain of β-catenin to its nuclear partner Lef1 have previously been used to mimic activation of Wnt/β-catenin signaling [29], [32], [44]. In particular, the strain of mice designated *Catnb^lox(Ex3)^*, in which exon 3 of the β-catenin gene has been flanked by loxP sites [44], has been extensively used to study the effect of activated Wnt/β-catenin signaling. Exon 3 encodes serine/threonine residues of β-catenin, which are targets of phosphorylation by GSK3 β kinase, the event that leads to β-catenin degradation. The product of Cre/loxP recombination in *Catnb^lox(Ex3)^* mice is the stabilized form of β-catenin, functionally equivalent to the delβ-CAT protein used here and elsewhere [32]. As the study presented here and that of [27] both employed stabilized forms of β-catenin, the difference in phenotypes must lie in the regulatory regions used. In case of β-catenin stabilization using the Cre/loxP system [27], β-catenin accumulation in the nucleus is the result of a two-step process. First, Cre recombinase is produced from the αA-crystallin promoter in lens fiber cells beginning at E13.5 (MLR39 Cre line). After recombination, transcription of the stabilized β-catenin is dependent on the activity of endogenous regulatory elements of the β-catenin gene in differentiating lens fiber cells. The level of β-catenin expression in lens fiber cells after this particular genetic manipulation is unclear, as no proof of stabilized β-catenin expression has been provided [27]. In contrast, the mouse αA-crystallin promoter is a reliable and frequently used regulatory element which allows medium-level expression of heterologous proteins in the lens fiber cell compartment [18], [34], [39], [45]–[48]. Alternative explanation of the different findings in our and Martinez et al. study [27], could be the phase of differentiation of manipulated fiber cells. Based on CLEF detection in αA-CLEF lenses, in the present study we have targeted early fiber cells (cells in transitional zone). Thus cells manipulated here were initiating the differentiation, whereas in previous study [27] only the population of fiber cells that have already initiated differentiation (outer cortical fiber cells) were affected.

Lens cataract is very often characterized by conversion of soluble crystallins into insoluble form [49], and this can be initiated by mutation in crystallins [50] or by changed physiological conditions inside the lens cells [49]. γ-Crystallins are specifically expressed in differentiating and differentiated lens fiber cells; moreover, their expression is associated with decreased mitotic activity of the fiber cell [2]. We have observed downregulation of γ-crystallins in αA-CLEF lenses. It is at present unclear if this is a direct effect of CLEF transcription factor or a consequence of an incomplete fiber cell differentiation in αA-CLEF lenses, although the second scenario is more likely. Most of the functional studies of γ-crystallin promoters have been performed with γD- and γF-crystallin promoters, however the direct regulation of γ-crystallin genes by Lef/Tcf family of transcription factors has not been described (reviewed in [51]). Moreover, we were unable to identify Lef/Tcf binding sites (sequence logo CTTTGAT) in the known regulatory regions of γD- and γF-crystallins (unpublished data). It was shown previously that transcription factor Pax6 can act as a transcriptional regulator of γF-crystallin promoter [52], [53]. Pax6 was detected in the more mature lens fiber cells (in the central part of the lens) of αA-CLEF mice. It is very well possible that Pax6 participates in the repression of γ-crystallin gene expression, thus contributing to the lower levels of γ-crystallin protein in adult αA-CLEF lenses.

Disorganized distribution of fiber cell nuclei in αA-CLEF embryonic lenses was the first indication of impaired differentiation of fiber cells. The persistence of fiber cell differentiation markers in the fiber cell compartment of transgenic αA-CLEF lenses suggested delayed differentiation of fiber cells, which was shifted from the transitional zone to the central part of the lens. This is reminiscent of the effect of β-catenin stabilization within the entire lens [27] that resulted, among other effects, in delayed differentiation of lens epithelial cells. Since Wnt/β-catenin signaling is known to regulate the cell cycle [42], we examined the expression of cell cycle promoting factors cyclin D1 and cyclin D2, and the expression of negative regulators of the cell cycle, cyclin-dependent kinase inhibitors p27^Kip1^ and p57^Kip2^. Cyclin D1 and cyclin D2 are closely related G1 cyclins [54]. Importantly, cyclin D1 is also a known target of the Wnt/β-catenin signaling pathway [55], [56]. We thus expected its altered expression pattern due to the Wnt/β-catenin signaling activation in lens fiber cells. Cyclin D1 and cylin D2 are normally expressed in proliferating lens epithelial cells and in equatorial lens fiber cells because they are required for G1 to S phase transitional, whereas their expression is absent from fiber cells that have already completed the differentiation [43], [46], [57]. As cyclin-dependent kinase inhibitor (Cdk) p27^Kip1^ is required together with p57^Kip2^ for inhibition of Cdk’s responsible for G1/S transition [11], p27^Kip1^ expression coincides with the cell cycle exit during the fiber cell differentiation [46]. Since expression of p27^Kip1^ and p57^Kip2^ in the transitional zone appears to require the activity of transcription factor Prox1 [4], aberrant expression of p27^Kip1^ at E16.5 in the central part of the lens may very well be due to the stronger Prox1 expression in this compartment in αA-CLEF lenses.

It was shown previously that the loss of β-catenin in the entire lens (epithelium and fiber cells) results in downregulation of cyclin D1 in lens epithelial cells, and at the same time in apparent upregulation of cyclin D1 in the more matured differentiating fiber cells [21], a somewhat surprisingly contradictory effect. It is well established that active Wnt/β-catenin signaling induces cyclin D1 and thereby triggers G1-progression [55], [56], preventing cell cycle exit to G_0_. It is therefore not surprising that activation of Wnt/β-catenin in fiber cells in αA-CLEF mice induces an increase of cyclin D1 and cyclin D2 in the more matured differentiating fiber cells (located in the central part of the lens). Analysis of R26p-Fucci2 mouse embryos at E13.5 provides convincing visual confirmation that lens epithelial cells are in S/G2/M phase, whereas differentiating fiber cells in the lens equatorial region are in G1 phase [58]. It is likely that the differentiating fiber cells can be kept in G1 by the aberrantly present cyclin D1 and cyclin D2, activated via Wnt/β-catenin signaling at E13.5 in αA-CLEF mice.

In summary, we have shown here that ectopic activation of Wnt/β-catenin signaling in lens fiber cells during embryonic development results in delayed fiber cell differentiation and cataract formation. A more direct link between the activated Wnt/β-catenin signaling in lens fiber cells during embryonic development and cataract formation in adulthood is still missing and needs deeper investigation of the changes in lens physiology in consequence of the changed molecular regulation of the lens development.

## Supporting Information

Figure S1 Eye phenotype of transgenic αA-CLEF founders. Histological sections of eyes stained with hematoxylin and eosin of adult wild-type (A) and three transgenic founders (B) MB06-01, (C) MB06-05, and (D) MB06-12 of the αA-CLEF mouse line. Note the disrupted lens morphology of transgenic lenses (B, C, D).
(TIF)

Figure S2 Expression of β- and γ-crystallin in αA-CLEF lenses. (A, B, C, D) Comparable β-crystallin expression was detected in E13.5 and E16.5 wild-type and αA-CLEF lenses, enlarged lens shown in (A′, B′). (E, F, G, H) Similarly, no difference in γ-crystallin expression was observed in E13.5 and E16.5 wild-type and αA-CLEF lenses, enlarged lens shown in (E′, F′). Scale bars indicate 50 µm.
(TIF)

Figure S3 ZO-1 and N-cadherin localization in αA-CLEF lenses at E13.5 and E16.5 is unchanged. (A-D) ZO-1 is strongly expressed in epithelium-fiber cell interface (arrowheads) in wild-type (A, C) and αA-CLEF lenses (B, D). No obvious difference in ZO-1 localization in αA-CLEF lenses is observed compared to wild-type mice. (E-H) N-cadherin is present in both epithelial and fiber cells in wild-type lenses (E, G) and there is no apparent difference in N-cadherin expression in αA-CLEF lenses (F, H). Scale bars indicate 50 µm (A, B, E, F) and 100 µm (C, D, G, H).
(TIF)

Figure S4 Epithelial-mesenchymal transition and apoptosis are not induced in αA-CLEF lenses. (A, C) Marker of epithelial-mesenchymal transition α-SMA is not present in E13.5 or in E16.5 wild-type lenses, but is present in the muscles of eyelids and the iris (arrowheads). (B, D) α-SMA is not present at E13.5 and at E16.5 in αA-CLEF lenses. (E-F) No cleaved caspase 3 (cCas3)-positive cells are detected in wild-type E13.5 and E16.5 (E, G) or in αA-CLEF E13.5 and E16.5 (F, H) lenses. Dotted background (*) in central lens region is the artifact of anti-cCas3 staining on paraffin sections. Scale bars indicate 50 µm (A, B, E, F) and 100 µm (C, D, G, H).
(TIF)

Table S1 List of primers used for qRT-PCR.
(XLSX)
