# Supplementary figures and images for: Ectopic Activation of Wnt/β-Catenin Signaling in Lens Fiber Cells Results in Cataract Formation and Aberrant Fiber Cell Differentiation
Source: PLoS One. 2013 Oct 30;8(10):e78279. doi: 10.1371/journal.pone.0078279 (PMC3813504; doi:10.1371/journal.pone.0078279)

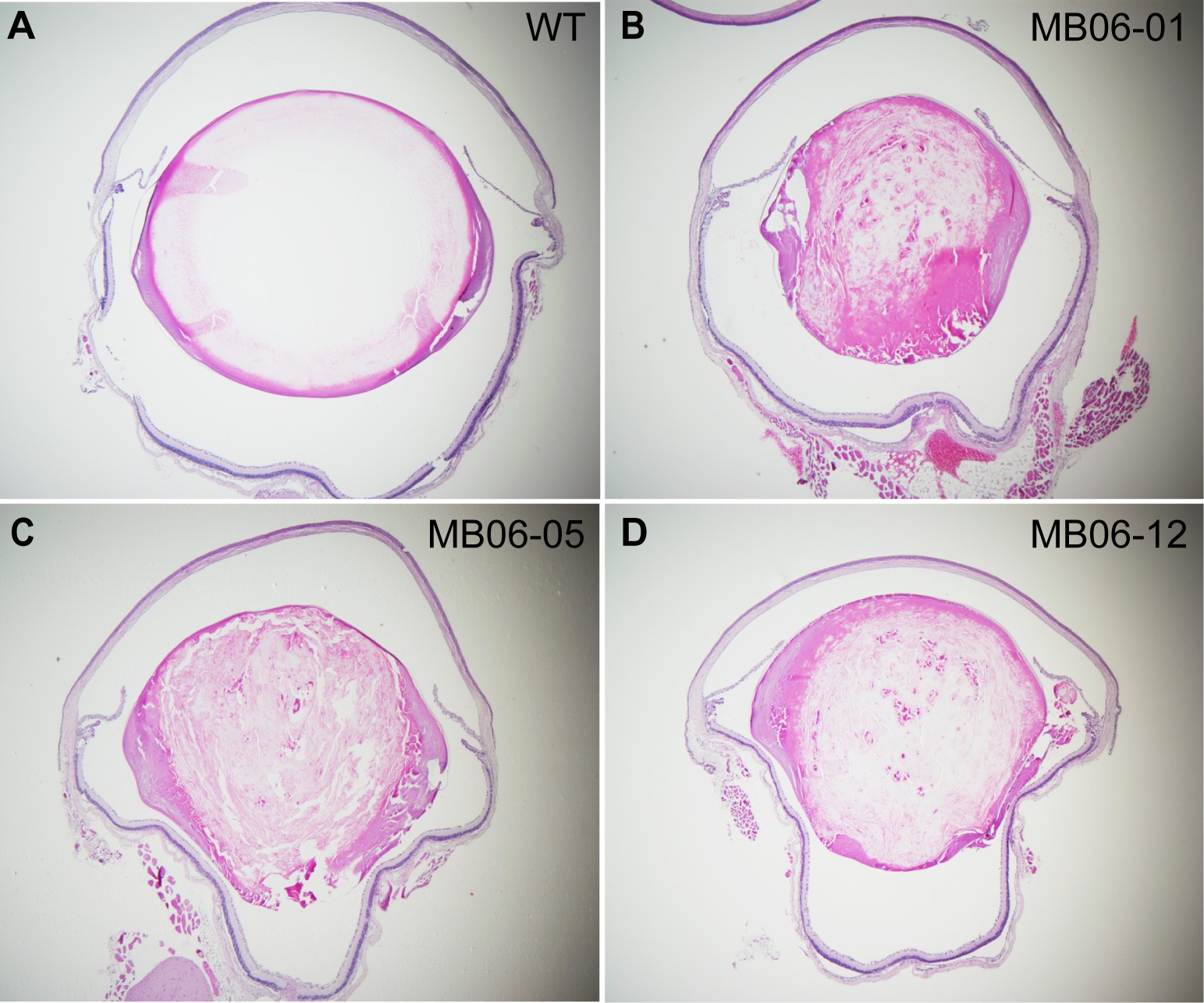

Supplement: Figure S1 — Eye phenotype of transgenic αA-CLEF founders. Histological sections of eyes stained with hematoxylin and eosin of adult wild-type (A) and three transgenic founders (B) MB06-01, (C) MB06-05, and (D) MB06-12 of the αA-CLEF mouse line. Note the disrupted lens morphology of transgenic lenses (B, C, D). (TIF) [file pone.0078279.s001.tif]

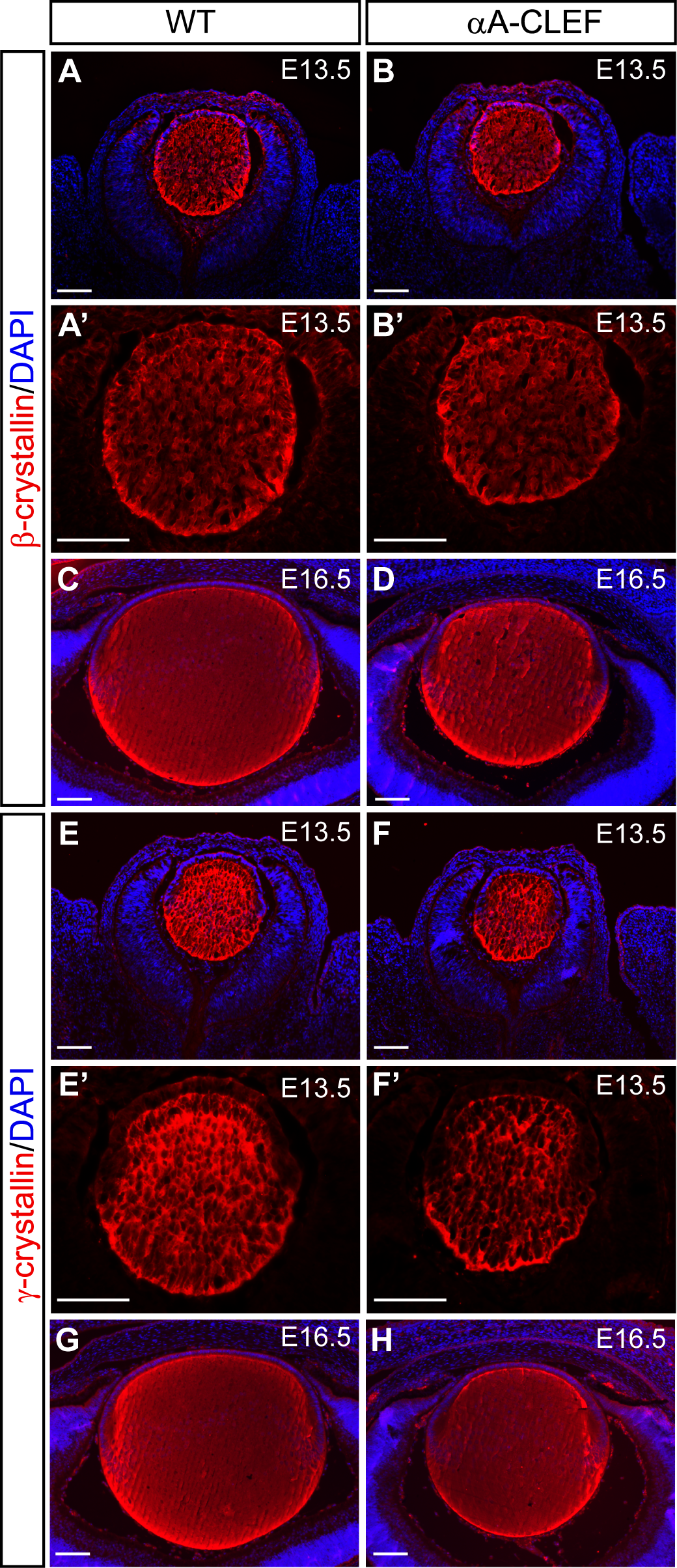

Supplement: Figure S2 — Expression of β- and γ-crystallin in αA-CLEF lenses. (A, B, C, D) Comparable β-crystallin expression was detected in E13.5 and E16.5 wild-type and αA-CLEF lenses, enlarged lens shown in (A′, B′). (E, F, G, H) Similarly, no difference in γ-crystallin expression was observed in E13.5 and E16.5 wild-type and αA-CLEF lenses, enlarged lens shown in (E′, F′). Scale bars indicate 50 µm. (TIF) [file pone.0078279.s002.tif]

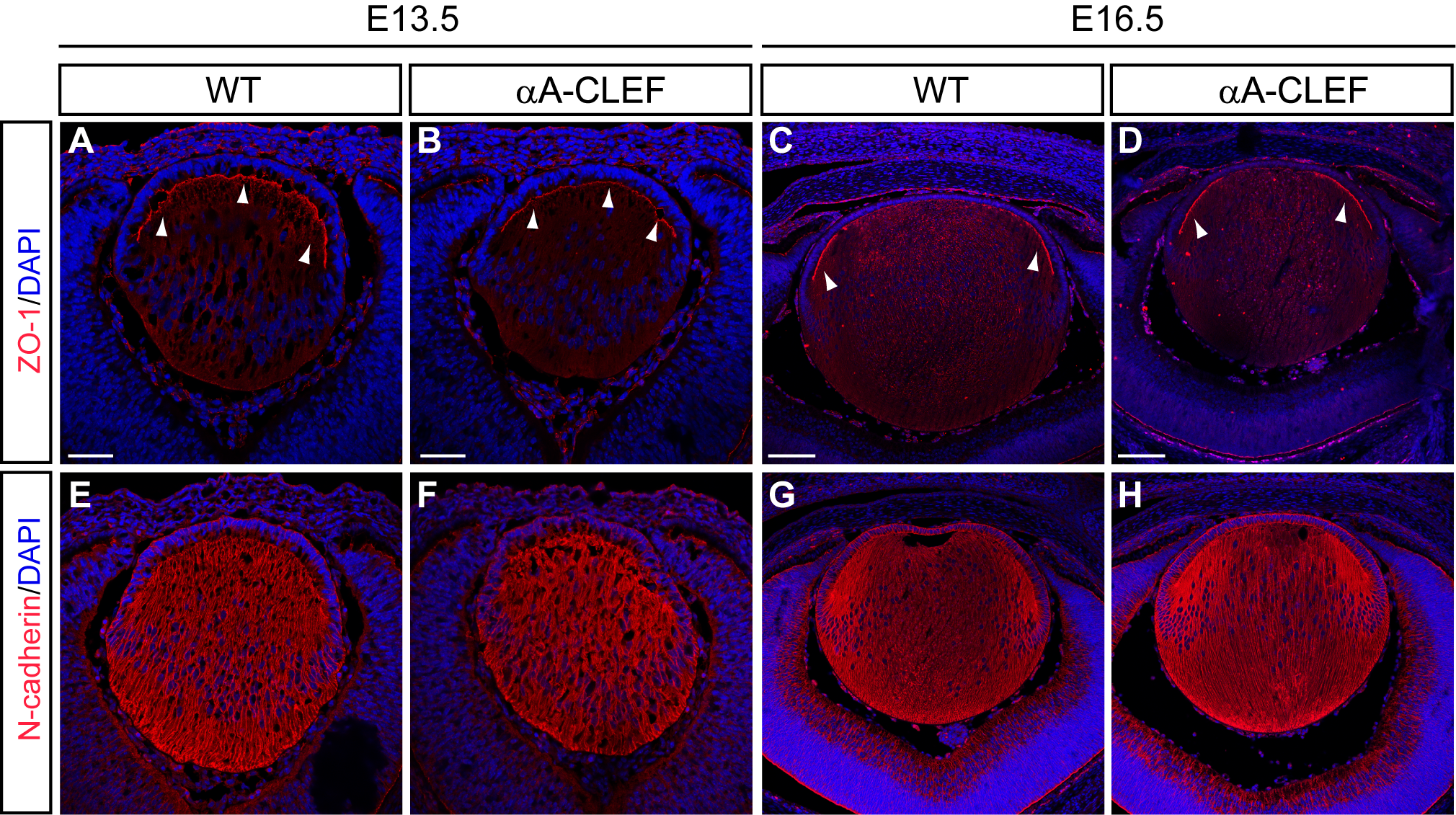

Supplement: Figure S3 — ZO-1 and N-cadherin localization in αA-CLEF lenses at E13.5 and E16.5 is unchanged. (A-D) ZO-1 is strongly expressed in epithelium-fiber cell interface (arrowheads) in wild-type (A, C) and αA-CLEF lenses (B, D). No obvious difference in ZO-1 localization in αA-CLEF lenses is observed compared to wild-type mice. (E-H) N-cadherin is present in both epithelial and fiber cells in wild-type lenses (E, G) and there is no apparent difference in N-cadherin expression in αA-CLEF lenses (F, H). Scale bars indicate 50 µm (A, B, E, F) and 100 µm (C, D, G, H). (TIF) [file pone.0078279.s003.tif]

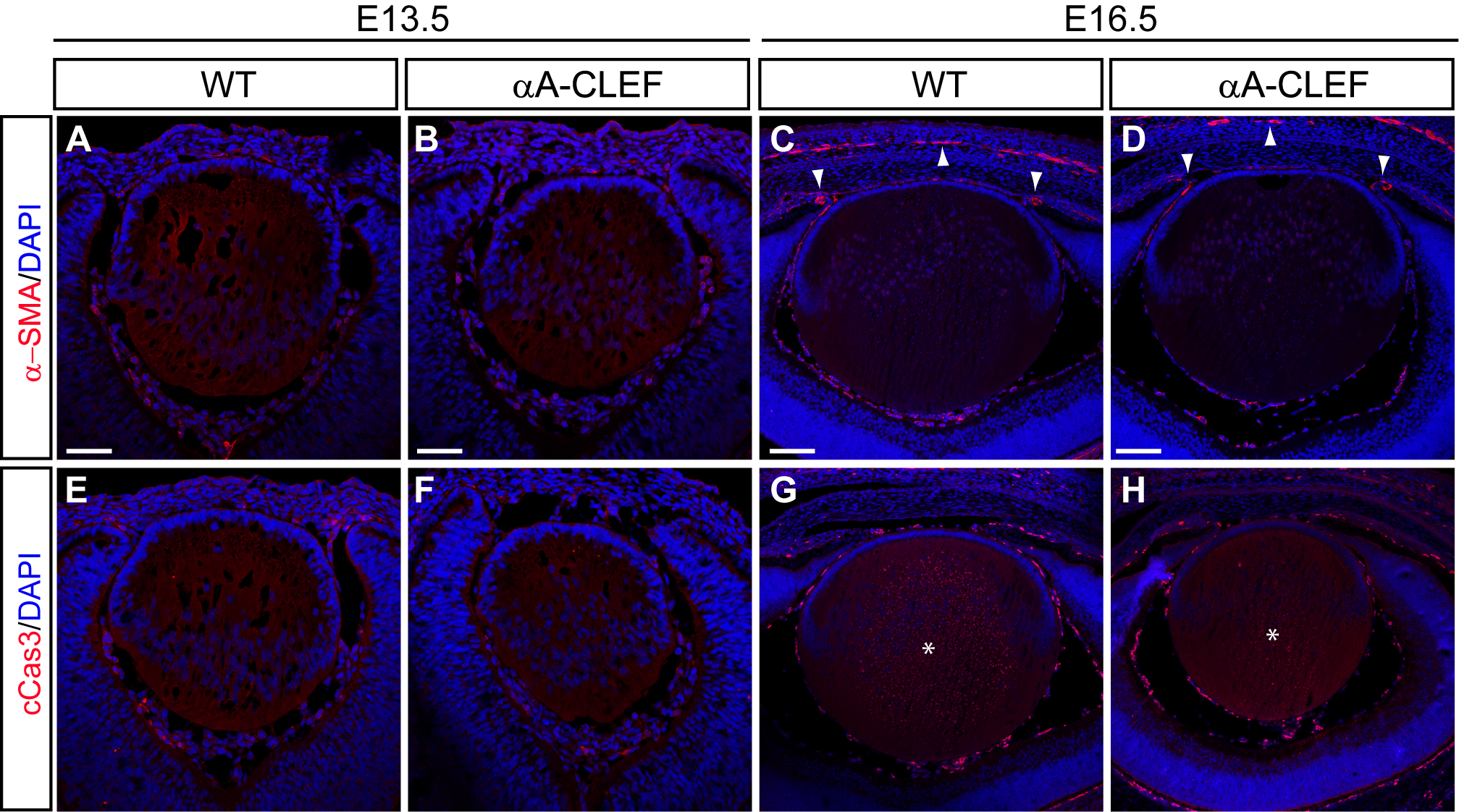

Supplement: Figure S4 — Epithelial-mesenchymal transition and apoptosis are not induced in αA-CLEF lenses. (A, C) Marker of epithelial-mesenchymal transition α-SMA is not present in E13.5 or in E16.5 wild-type lenses, but is present in the muscles of eyelids and the iris (arrowheads). (B, D) α-SMA is not present at E13.5 and at E16.5 in αA-CLEF lenses. (E-F) No cleaved caspase 3 (cCas3)-positive cells are detected in wild-type E13.5 and E16.5 (E, G) or in αA-CLEF E13.5 and E16.5 (F, H) lenses. Dotted background (*) in central lens region is the artifact of anti-cCas3 staining on paraffin sections. Scale bars indicate 50 µm (A, B, E, F) and 100 µm (C, D, G, H). (TIF) [file pone.0078279.s004.tif]
